# Genomic DNA Hypomethylation Is Associated with Neural Tube Defects Induced by Methotrexate Inhibition of Folate Metabolism

**DOI:** 10.1371/journal.pone.0121869

**Published:** 2015-03-30

**Authors:** Xiuwei Wang, Zhen Guan, Yan Chen, Yanting Dong, Yuhu Niu, Jianhua Wang, Ting Zhang, Bo Niu

**Affiliations:** 1 Department of Biochemistry and Molecular Biology, Shanxi Medical University, Taiyuan, China; 2 Beijing Municipal Key Laboratory of Child Development and Nutriomics, Capital Institute of Pediatrics, Beijing, China; 3 Department of Respiratory, the Second Hospital of Shanxi Medical University, Taiyuan, China; University of Florida, United States

## Abstract

DNA methylation is thought to be involved in the etiology of neural tube defects (NTDs). However, the exact mechanism between DNA methylation and NTDs remains unclear. Herein, we investigated the change of methylation in mouse model of NTDs associated with folate dysmetabolism by use of ultraperformance liquid chromatography tandem mass spectrometry (UPLC/MS/MS), liquid chromatography-electrospray ionization tandem mass spectrometry (LC-MS/MS), microarray, matrix-assisted laser desorption/ionization time-of-flight mass spectrometry and Real time quantitative PCR. Results showed that NTD neural tube tissues had lower concentrations of 5-methyltetrahydrofolate (5-MeTHF, *P* = 0.005), 5-formyltetrahydrofolate (5-FoTHF, *P* = 0.040), S-adenosylmethionine (SAM, *P* = 0.004) and higher concentrations of folic acid (*P* = 0.041), homocysteine (Hcy, *P* = 0.006) and S-adenosylhomocysteine (SAH, *P* = 0.045) compared to control. Methylation levels of genomic DNA decreased significantly in the embryonic neural tube tissue of NTD samples. 132 differentially methylated regions (35 low methylated regions and 97 high methylated regions) were selected by microarray. Two genes (*Siah1b*, *Prkx*) in Wnt signal pathway demonstrated lower methylated regions (peak) and higher expression in NTDs (*P*<0.05; *P*<0.05). Results suggest that DNA hypomethylation was one of the possible epigenetic variations correlated with the occurrence of NTDs induced by folate dysmetabolism and that *Siah1b*, *Prkx* in Wnt pathway may be candidate genes for NTDs.

## Introduction

Neural tube defects (NTDs) are severe congenital malformations in the central nervous system. It has been reported that 50%~70% of NTDs in human could be prevented by the periconceptional consumption of folic acid[1–3]. However, the exact mechanism between folate deficiency and NTDs remains unclear.

Folic acid is converted to active tetrahydrofolate by dihydrofolate reductase (DHFR) and then participates in the process of one-carbon unit transfer. This process plays a critical role in the methylation of DNA[4]. Two times epigenetic reprogramming was occurred in early embryonic development, including rapid demethylation and re-methylation, to remove parental genomic imprinting and establish the embryonic DNA methylation patterns. When folate deficiency was caused by various factors in embryogenesis, the methylation patterns of normal embryonic development may be changed, resulting in birth defects. In the previous study, we found that methylation of long interspersed nucleotide element-1(LINE-1) may be a potential indicator for global DNA methylation, and hypomethylation of LINE-1 and genomic DNA was associated with an increased risk for NTDs [5]. Folate deficiency affected the homeostasis of folate-mediated one-carbon metabolism, leading to reduced LINE-1 methylation in mouse embryonic stem cells (ESCs) [6]. Copp [7] et al reported that the incidence of NTDs increased when the methylation cycle were inhibited in mouse and chick animal models. Waterland[8] et al showed that dietary methyl supplementation with extra folic acid, vitamin B (12), choline, and betaine altered the phenotype of their offspring via increased CpG methylation. DNA methyltransferase 1(*DNMT1*) plays an important role in the inheritance of genomic DNA methylation. Early embryonic lethality in *DNMT1*-null mutant mice indicates that DNA methylation is essential for mammalian embryonic development [9]. Animal studies and human data on the imprinted *IGF2* (insulin-like growth factor 2) locus indicated a link between prenatal nutrition and DNA methylation, and persistent changes in DNA methylation may be a common consequence of prenatal famine exposure [10]. Above all, the change in DNA methylation pattern caused the disorder of development, leading to birth defects. However, abnormal methylation in NTDs with folate-associated dysmetabolism has not been reported.

This study investigated the mechanism of methylation changes in methotrexate (MTX) induced NTDs for the first time, which is of great importance to elucidate the exact mechanism between folate deficiency and NTDs.

## Results

### MTX-Induced NTDs and Embryonic Abnormalities

All embryos were examined carefully using a dissecting microscope. MTX caused a NTD incidence of 30.8%. The details of the types of other abnormalities and the folate-associated dysmetabolisms caused by MTX have been described previously [11, 12].

### Concentrations of folates in embryonic tissues

To confirm the effect of MTX injection, folate contents were detected by UPLC/MS/MS. The concentrations of 5-methyltetrahydrofolate (5-MeTHF), 5-formyltetrahydrofolate (5-FoTHF) and S-adenosylmethionine (SAM) in embryonic neural tube tissues were significantly decreased (*P*<0.05) and the concentrations of folic acid, homocysteine (Hcy), S-adenosylhomocysteine (SAH) were significantly increased compared to control embryonic neural tissues (*P*<0.05) (Table 1).

**Table 1:** Folate contents concentrations in control and NTDs from neural tube tissues(μg g^-1^ protein).

	Control	NTDs	*P*
Folic acid	2.73±1.03	4.40±1.38	0.041
5-MeTHF	9.55±2.49	4.61±2.19	0.005
SAM	10.56±4.13	5.92±1.83	0.040
SAH	3.55±1.27	5.61±1.77	0.045
5-FoTHF	4.94±0.88	3.21±0.72	0.004
Hcy	4.275±1.36	7.09±1.41	0.006

### DNA hypomethylation in NTDs

**Fig 1:**
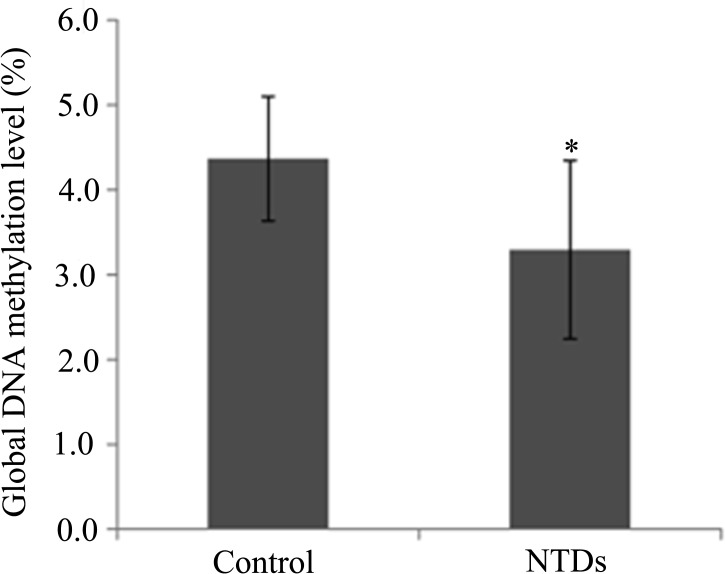
Methylation levels in NTDs and control groups. *P<0.05 NTDs versus control.

To determine whether DNA methylation was affected by folate metabolic disorder, we measured the global DNA methylation in neural tube tissues from control and NTDs by Liquid chromatography-electrospray ionization tandem mass spectrometry (LC-MS/MS). We observed that neural tube tissues had significantly decreased global DNA methylation levels compared to control (*P*<0.05) (Fig. 1).

### Identification of differentially methylated genes

**Fig 2:**
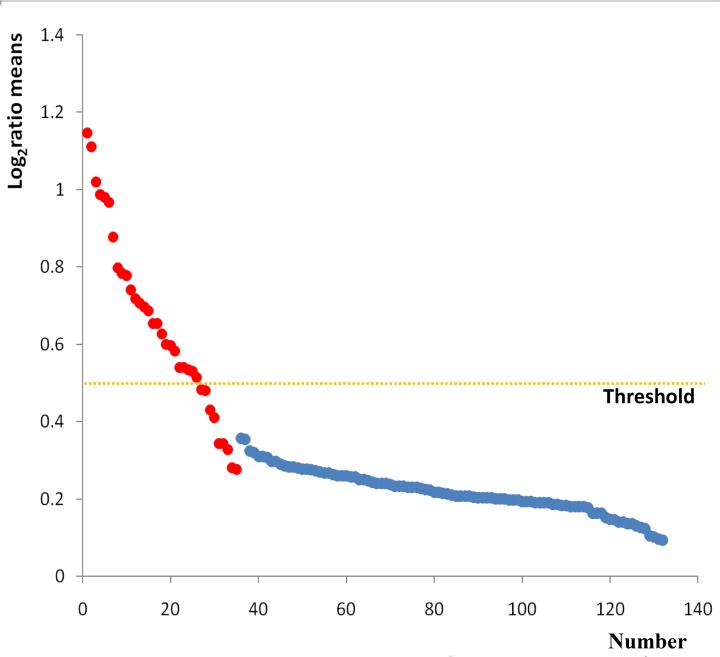
Log2 ratio means of peaks and gene annotation. Log2 ratio means: all probe average of peak area of three NTD samples, red dot represents the area of low methylation NTDs vs control, blue dot represents of high methylation NTDs vs control. The threshold line is the cutoff value, as the log2 ratio means values = 0.5. The numbers on the horizontal axis indicate the sorting number of genes. We did the sorting according to the numerical value of log2 ratio means. The sorting number of related genes showed as S1 Fig.

Samples in each group had three biological replicates. NimbleScan software was used to screen the differentially methylated regions. According to the criterion of cutoff limitation that Minimum Probes per peak is 2, *P*-value minimum cutoff (-log10) is 2, Maximum spacing between nearby probes within peak (bp) is 500bp, Sliding window width (bp) is 750. A total of 132 differentially methylated regions (35 low methylated regions and 97 high methylated regions) were selected from the two groups. Log_2_Ratio means value showed the methylated fold change between the two groups. The greater the Log2 ratio means value, the higher the credibility of the results. In our study, differentially methylated genes were showed in the Fig. 2, log2 ratio means values are relatively large in hypomethylation (red dots), the maximum is 1.1467, the minimum is 0.2767, and log2 ratio means values are relatively small in hypermethylation (blue dots), the maximum is 0.3567, the minimum is 0.0933. When the log2 ratio means values ≥0.5, the altered methylated region was considered as a high reliability change. So we only selected the log2 ratio means values ≥0.5 as the candidate peak for further analysis, and 26 peaks including 30 genes were selected in low methylated regions and found on Chromosome X.

**Fig 3:**
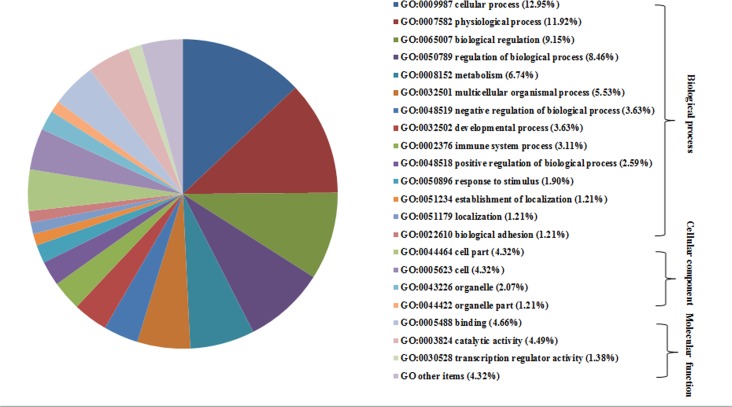
GO categories of the hypomethylation genes. GO categories for the hypomethylation genes. The genes were classified into biological processes, cellular component, and molecular function.

**Fig 4:**
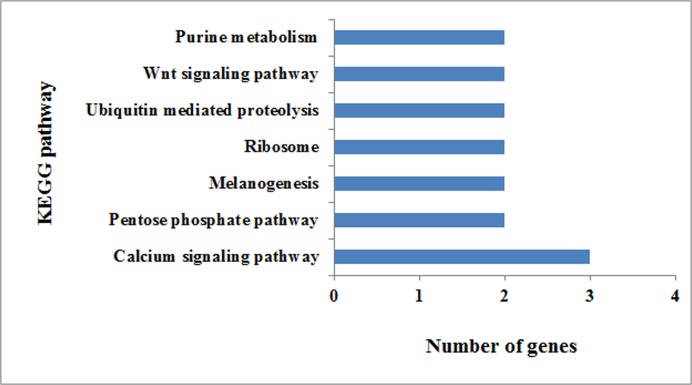
KEGG pathways of the low methylation genes. Those pathways involving more than two genes were selected for mapping and are listed according to the number of genes.

The selected genes were then analysed in the context of GO analysis using MAS3.0 (http://bioinfo.capitalbio.com/mas3/). As shown in Fig. 3, they are divided into three categories including biological processes, molecular function and cellular components. The molecular function category mainly consisted of three subcategories: catalytic activity, binding and transcription regulator activity. In the biological process category, genes related to cellular process, metabolism and developmental process were found. KEGG analysis of the selected genes was also performed to identify potential novel candidates that are thought to be related to the NTD characteristics. After mapping the selected genes to KEGG, 42 pathways were involved (Fig. 4). Wnt signaling pathway plays an important role in the development of neural tube, *Siah1b*, *Prkx* in Wnt signaling pathway were prioritized.

### Effect of MTX on Gene methylation

**Fig 5:**
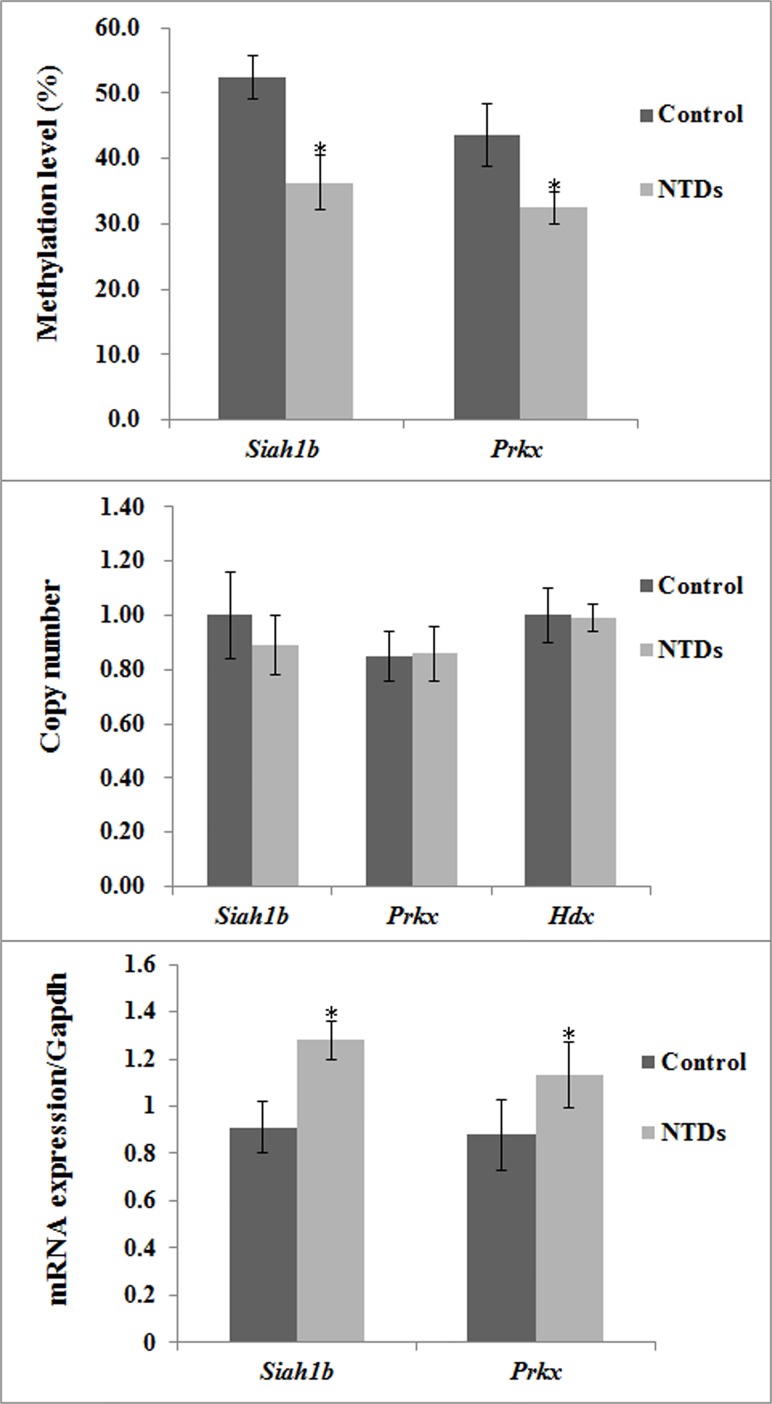
Confirmation of the differential methylation genes. A:Changes in DNA methylation levels in *Siah1b* (ChrX:160513241–160516941), *Prkx* (ChrX:75040559–75044259); B: Comparisons of the two genes expressions between NTDs and control samples; C: Comparisons of copy number varitions of the two genes between NTDs and control samples, *P<0.05 NTDs versus control.

In the previous microarray study, we found the hypomethylation of *Siah1b* (ChrX:160513241–160516941), *Prkx* (ChrX:75040559–75044259). To confirm this results, we measured methylation level of *Siah1b* (ChrX:160513241–160516941), *Prkx*(ChrX:75040559–75044259) promoter sequences in embryonic neural tube tissues from both NTDs and control samples. Those sequences were amplified from 3 NTDs and 3 control samples after bisulfite conversion of genomic DNA. The mean methylation levels were then compared by using Student’s T test. The methylation levels in the NTD samples were significantly lower than that in the control samples (Fig. 5A, *P*<0.05).The results were consistent with microarray assays.

Furthermore, we performed quantitative real time PCR to compare the copy numbers of *Siah1b*, *Prkx* genes. Our results showed that there was no differences between NTDs and control (Fig. 5B, *P*>0.05).

To determine whether the abnormal *Siah1b*, *Prkx* methylation levels results in the expression of two genes, quantitative real time PCR was used to detect their expressions. We found the expression levels of the two genes were higher in NTDs than control (Fig. 5C, *P*<0.05).

## Discussion

In this study, we observed the folate dysmetabolism and global DNA hypomethylation in embryonic neural tube tissues of NTDs. Furthermore, microarray was used to identify differentially methylated genes in the embryonic neural tissues of NTDs induced by MTX associated with folate-dysmetabolism in pregnant mice compared to controls. A total of 132 differentially methylated regions (35 low methylated regions and 97 high methylated regions) were selected from the two groups. Genes in Wnt pathway (*Siah1b*, *Prkx*) were confirmed by quantitative real time PCR. These two genes might contribute to the pathogenesis of MTX-induced NTDs on the basis of hypomethylation at the epigenetic level.

MTX is a specific inhibitor to DHFR, an essential enzyme in the folate metabolic pathway, leading to the failure of folate being reduced to tetrahydrofolate and thus folate-related dysmetabolism. In our study, we injected MTX into pregnant mice, which resulted in a decreased level of one-carbon unit in the embryonic neural tube tissues of NTDs. The concentrations of 5-MeTHF, 5-FoTHF, and SAM were significantly decreased, and the concentration of Hcy and SAH was increased significantly. These results further support the notion of a close association between folate metabolic disorders and NTDs.

Aberrations in genome-wide methylation during embryogenesis have been linked to developmental abnormalities at birth[13]. Several reports showed that inhibition of the methylation cycle induced a high frequency of cranial NTDs in cultured chick and mouse embryos [14–16]. Those suggest that aberrant genomic methylation underlies the complex pathogenesis of NTDs. In this study, we found that global methylation levels were significantly lower in embryonic neural tissues of NTDs. DNA hypomethylation increases recombination and/or hamper chromosomal segregation during mitosis[5, 17], which may play a role in the development of chromosome abnormalities in NTDs and shed some light on the mechanisms involved in the cause of NTDs.

DNA methylation is an epigenetic modification, which play an important role in maintaining chromosome structure, X chromosome inactivation, gene imprinting and tumor development[18]. In the embryonic development at E3.5 day, the overall methylation level begin to increase, and achieve higher level at E6.5 day. The higher methylation level was maintained until birth [19]. The methylation level of the promoter CpG islands may affect the gene transcription regulation, and make the gene silence. It has been reported that methylation is involved in X chromosome inactivation [20]. In this study, we screened the differential methylated regions by the NimbleGen mouse DNA methylation microarray. Results showed that 35 low methylation regions and 97 high methylation regions were found. When the log2 ratio means values ≥0.5, 26 peaks including 30 genes were selected in low methylated regions. The altered methylated region was considered as a high reliability change. The selected genes were then analysed in the context of GO biological process using MAS3.0 to select the NTD candidate genes. 2 genes in Wnt pathway were selected as good candidates for further investigation. The 2 genes expression profiling and copy number were detected by real time quantitative PCR which showed that the 2 genes exhibited upregulated expression in NTD embryonic neural tissues compared to that in control embryonic neural tissues. Besides, the copy numbers of *Siah1b*, *Prkx* genes showed no difference between NTDs and control embryonic neural tissues. Wnt signaling pathway plays an important role in the development of neural tube. It’s not only involved in the formation of dorsal and ventral axis of embryo, but in the developmental events of the establishment of cell polarity, cell fate decisions et al. *Siah1b* is one of the seven in absentia homolog (*Siah*) family. Overexpression of *Siah1* can mimic the effects of p53 activation and induce cell cycle arrest or apoptosis [21–23]. The expression of *Siah1b* is induced by p53 during apoptosis and tumor reversion, and the *Siah1b* gene is a direct transcriptional target of p53 [24, 25]. *Prkx* encodes a serine threonine protein kinase that has similarity to the catalytic subunit of cyclic AMP dependent protein kinases, and regulates cell proliferation, differentiation, and migration[26], and *Prkx* has been suggested to play important roles in neuronal differentiation [27]. Therefore, we speculate that the change of *Siah1b*, *Prkx* genes methylation give rise to the abnormal expression of *Siah1b*, *Prkx*, eventually lead to NTDs. However, the exact effects of the 2 candidate genes need to be studied further and may offer insights into the mechanisms underlying NTDs.

In conclusion, hypomethylation of genomic DNA and the genes (*Siah1b*, *Prkx*) in Wnt signal pathway is one of the possible epigenetic variations associated with the complex etiology of NTDs. Folate and its related dysmetabolisms influence the process of genomic DNA methylation. Abnormal methylation caused the change of gene expression, induced cellular misbehavior, including apoptosis, proliferation and differentiation, led to the occurrence of NTDs. In this study, it provided the preliminary theoretical and experimental basis to further reveal the mechanism of NTDs induced by folic acid metabolic disorder. Howerver, there was limitation for the mice model of NTDs. As results showed in our study, 20% of murine embryos were resorbed following MTX treatment at 4.5mg/kg body weight [12]. This means that the fetuses we examined from the MTX-treated mothers represented a select subset of all the fetuses, which may bias ours results. The exact mechanism concerning the relationship of abnormal methylation with NTDs induced by folate dysmetabolism needs to be further clarified.

## Materials and Methods

### Establishment of NTD mice model by MTX

All experimental procedures were reviewed and approved by the Animal Ethics Committee of the Capital Institute of Pediatrics (CIP2009012). NTD mice model was established by intraperitoneal injection of MTX (4.5mg/kg body weight) on gestational day 7.5 (GD7.5) into pregnant mice according to our previous study [11, 12]. MTX injection induced no significant weight loss in the mother compared to control on gestational day 11.5 when they are sacrificed. All surgery was performed under sodium pentobarbital anesthesia, and all efforts were made to minimize suffering of mice. The mice were sacrificed by anesthetic overdose. Embryonic neural tissues from the most rostral aspect of the forebrain to the caudal aspect of the hindbrain (above the otic vesicle) were microdissected and checked to eliminate any mesoderm or non-neural tissues as precisely as possible. The location from which the sample of the neural tube was obtained was consistent in all fetuses as precisely as possible. The sample of neural tube was obtained from the animals with exencephaly and without other malformation under a dissect microscope.

### Detection of folate contents in embryonic tissues

Embryonic tissue samples were homogenized with extraction buffer (0.05mol/LMES,0.1mol/L DET, 1% sodium ascorbate, pH6.0), heat extracted at 100°C for 15min and centrifuged for 15min at 36000×g. Conjugase from folate-free rat plasma was added to the supernatant and incubated at 37°C for 1.5h. The samples were then heated at 100°C for 5min, and centrifuged for 20 min at 36000×g [28]. The supernatant were detected using UPLC/MS/MS methods following the previous description[29].

### DNA extraction

Genomic DNA was extracted from three control and three NTD samples, respectively, according to manufacturer’s instructions of DNeasy Blood & Tissue Kit (QIAGEN, Germany). The genomic DNA should be undegraded and have A260/A280≥1.8 and A260/A230≥1.9 according to NimbleGen quality control requirements.

### Global methylation analyses by LC-MS/MS[30]

An Agilent tandem mass spectrometer (G6410B) was used for LC-MS/MS analysis. Separation was performed on an Aglient ZORBAX SB-AQ C18 column (2.1×100 mm, 3.5μm). The mobile phases were set as follows: methanol (phase A) and water (phase B), both with 0.1% formic acid. The following linear elution gradient was used (flow rate, 0.2 ml/min): 0–2min, 100%A to 95%A; 2–2.5min, 95%A to 90%A, 2.5–3.5min, 90%A to 75%A; 3.5–7.5min, 75%A to 100%A, 7.5–10min, equilibration with 100%A. The total analysis time was 10 min and the injection volume was 10μL. The MS/MS phase of analysis was performed under positive-ion (ESI) mode. The parameters for ESI source were a gas temperature of 350°C, dry gas flow of 12 L/min, nebulizer pressure of 50 psi, and capillary voltage of 4000V. High purity nitrogen (purity>99.999%) was used as collision gas and ordinary nitrogen was used as nebulizing gas. Quantification of the deoxyribonucleosides was accomplished in multiple reactions monitoring mode (MRM).

### DNA methylation analyses using DNA methylation microarrays

The microarray analysis for six extracted DNA samples was performed using NimbleGen mouse DNA methylation 3×720K CpG islands plus refseq promoter arrays according to the manufacturer’s instructions (http://www.nimblegen.com/support/dna-microarray-support.html).

### Methylation measurement and analysis

Genomic DNA from NTD and control samples were treated with sodium bisulfite and purified by using the Wizard DNA Clean-Up System. The quality of the bisulfite conversion was controlled using PCR products without methylation.

The Sequenon MassARRAY platform was used to analyze the quantitative methylation according to the previous study [5](S1 Table).

### Confirmation Using Quantitative Real-time PCR

To confirm the microarray result, we used the same genomic DNA as in the microarray analysis for quantitative real-time PCR. Experiments were performed on an ABI prism7900HT sequence detection system (Applied Biosystems, Foster city, CA) using Power SYBR Green. The primers were designed using Primer 3.0 (http://frodo.wi.mit.edu/primer3) (S2 Table). Each assay was performed in triplicate using 20μL reactions containing 10μL 2×Power SYBR Green PCR Master Mix (Applied Biosystems), 200nM forward and reverse primers, and 20ng genomic DNA. Melting curve and sequence analysis were performed to verify PCR product specificity. The relative Ct method was used to quantify the copy number in NTD versus control samples. The Ct values for each set of triplicates were averaged and normalized to glyceraldehyde 3-phosphate dehydrogenase. The relative copy number for each sample was calculated using the 2^▲▲Ct^ method.

Relative mRNA expression levels were determined by SYBR Green I kit (Biotechs,Changchun,China) according to the following conditions:1 cycle at 95°C for 10min, 50cycles at 95°C for 15s and 60°C for 1min.

Data reports were similar as those described above (S3 Table).

### Statistical analyses

Data were analyzed using the SPSS 16.0 software package (McGraw-Hill Inc., New York, NY). Folate contents concentrations were expressed as mean±standard deviation (s.d.) and examined by one-factor analysis of variance (ANOVA). The results of quantitative real-time PCR were the average of triplicate reactions. Comparisons of copy numbers and gene expressions between control and NTD neural tissue samples were performed using Student’s t-tests and expressed as mean±standard deviation (s.d.). All P values were 2-sided, and *P*<0.05 was considered to be significant.

## Supporting Information

S1 Fig The sorting number of related genes. (TIF)

S1 File NC3Rs ARRIVE Guidelines Checklist (fillable).pdf. Animal Research: Reporting In Vivo Experiments.
(PDF)

S1 Table Primers for Methylation analysis. (DOC)

S2 Table Primers for copy number analysis. (DOC)

S3 Table Primers for mRNA analysis. (DOC)
