# Supplementary figures and images for: Genomic DNA Hypomethylation Is Associated with Neural Tube Defects Induced by Methotrexate Inhibition of Folate Metabolism
Source: PLoS One. 2015 Mar 30;10(3):e0121869. doi: 10.1371/journal.pone.0121869 (PMC4379001; doi:10.1371/journal.pone.0121869)

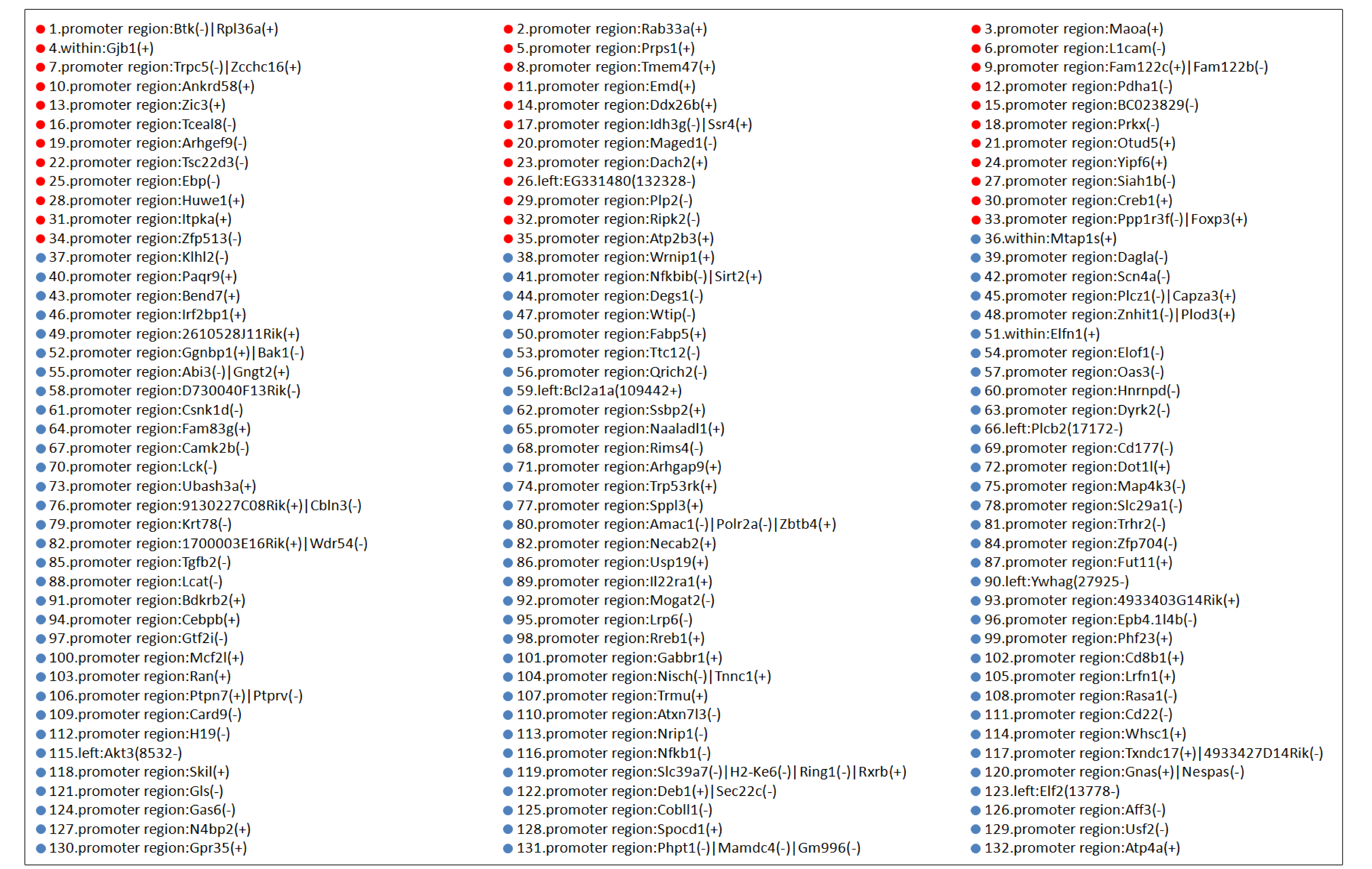

Supplement: S1 Fig — (TIF) [file pone.0121869.s001.tif]
