# Supplementary material for: Genomic DNA Hypomethylation Is Associated with Neural Tube Defects Induced by Methotrexate Inhibition of Folate Metabolism
Source: PLoS One. 2015 Mar 30;10(3):e0121869. doi: 10.1371/journal.pone.0121869 (PMC4379001; doi:10.1371/journal.pone.0121869)
Supplement: S1 Table — (DOC) [file pone.0121869.s003.doc]

S1 Table. Primers for Methylation analysis.

| **Gene** | **Primer** |
| --- | --- |
| *Siah1b* | 5’-aggaagagagGTGTAAAGGTTAAAGGGGTAGTTTT-3’ |
|  | 5’-cagtaatacgactcactatagggagaaggctAAATCAAAAACCTAAACAAATCACTT-3’ |
| *Prkx* | 5’-aggaagagag GGTTTAGTAATGAGAGTGTGGGAGATA-3’ |
|  | 5’-cagtaatacgactcactatagggagaaggct CCCACTACTAATACACAAACTACCA-3’ |
