# Supplementary material for: Genomic DNA Hypomethylation Is Associated with Neural Tube Defects Induced by Methotrexate Inhibition of Folate Metabolism
Source: PLoS One. 2015 Mar 30;10(3):e0121869. doi: 10.1371/journal.pone.0121869 (PMC4379001; doi:10.1371/journal.pone.0121869)
Supplement: S2 Table — (DOC) [file pone.0121869.s004.doc]

S2 Table. Primers for copy number analysis.

| **Gene** | **Forward Primer** | **Reverse primer** |
| --- | --- | --- |
| *Siah1b* | AACGAAAGAGGGGACGTGAC | CTCCATCCGTCGCTTAGACC |
| *Prkx* | ATAGGCAGTTCTATGCCGCC | GCTGCTTTTGCCTTACCACC |
| *Hdx* | CTTGCTCTGAAAGAGGGCGA | CAGAGTGTTTATGCGCAGGC |
