# Supplementary material for: Genomic DNA Hypomethylation Is Associated with Neural Tube Defects Induced by Methotrexate Inhibition of Folate Metabolism
Source: PLoS One. 2015 Mar 30;10(3):e0121869. doi: 10.1371/journal.pone.0121869 (PMC4379001; doi:10.1371/journal.pone.0121869)
Supplement: S3 Table — (DOC) [file pone.0121869.s005.doc]

S3 Table. Primers for mRNA analysis.

| **Gene** | **Forward Primer** | **Reverse primer** |
| --- | --- | --- |
| *Siah1b* | GGAGACCTATGGGACACCCT | CATTAAGGGCTGGCTACTGG |
| *Prkx* | TGTCTGGGTTTCCCCCATTTT | GCTTGATGTCTTCTGCCCCA |
| *Gapdh* | AGAGGGATGCTGCCCTTACC | ATCCGTTCACACCGACCTTC |
